# Design, synthesis, in silico and in vitro antimicrobial screenings of novel 1,2,4-triazoles carrying 1,2,3-triazole scaffold with lipophilic side chain tether

**DOI:** 10.1186/s13065-017-0347-4

**Published:** 2017-11-21

**Authors:** Mohamed Reda Aouad, Mariem Mohammed Mayaba, Arshi Naqvi, Sanaa K. Bardaweel, Fawzia Faleh Al-blewi, Mouslim Messali, Nadjet Rezki

**Affiliations:** 1 Department of Chemistry, Faculty of Science, Taibah University, Al-Madinah Al-Munawarah, 30002 Saudi Arabia; 2 Department of Chemistry, Faculty of Sciences, University of Sciences and Technology Mohamed Boudiaf, Laboratoire de Chimie et Electrochimie des Complexes Metalliques (LCECM) USTO-MB, P.O. Box 1505, El M‘nouar, 31000 Oran, Algeria; 3 Department of Pharmaceutical Sciences, Faculty of Pharmacy, University of Jordan, 11942 Amman, Jordan

**Keywords:** Click chemistry, 1,2,3-triazole-1,2,4-triazole hybrids, Lipophilic side chain, Antimicrobial activity, Molecular docking

## Abstract

**Background:**

1,2,4-Triazoles and 1,2,3-triazoles have gained significant importance in medicinal chemistry.

**Results:**

This study describes a green, efficient and quick solvent free click synthesis of new 1,2,3-triazole-4,5-diesters carrying a lipophilic side chain via 1,3-dipolar cycloaddition of diethylacetylene dicarboxylate with different surfactant azides. Further structural modifications of the resulting 1,2,3-triazole diesters to their corresponding 1,2,4-triazole-3-thiones via multi-step synthesis has been also investigated. The structures of the newly designed triazoles have been elucidated based on their analytical and spectral data. These compounds were evaluated for their antimicrobial activities. Relative to the standard antimicrobial agents, derivatives of 1,2,3-triazole-bis-4-amino-1,2,4-triazole-3-thiones were the most potent antimicrobial agents with compound **7d** demonstrating comparable antibacterial and antifungal activities against all tested microorganisms. Further, the selected compounds were studied for docking using the enzyme, Glucosamine-6-phosphate synthase.

**Conclusions:**

The in silico study reveals that all the synthesized compounds had shown good binding energy toward the target protein ranging from − 10.49 to − 5.72 kJ mol^−1^ and have good affinity toward the active pocket, thus, they may be considered as good inhibitors of GlcN-6-P synthase.

## Background

The synthesis of 1,2,4-triazoles has become one of the most hot and popular topic in modern heterocyclic chemistry due to their various uses. In fact, 1,2,4-triazoles have gained considerable importance in medicinal chemistry due to their potential antimicrobial [1], anticancer [2], antitubercular [3], anticonvulsant [4] and anti-inflammatory [5] properties. In addition, several well know antifungal drugs including Fluotrimazole, Ribavirine, Fluconazole, Estazolam, Alprazolam and Loreclezole [6, 7] were found to possess the 1,2,4-triazole moiety in their structures.

The 1,2,3-triazole nucleus has been also recognized as a fascinating scaffold in drug design due to its incorporation into many chemotherapeutic drug molecules as antibacterial [8], anticancer [9], antifungal [10], antiviral [11] and antimalarial [12], antimycobacterial [13] agents.

Surfactants are widely studied by researchers due to their promising chemical, industrial and biological applications. Surfactants are associated with diverse biological properties such as antimicrobial [14], anti-inflammatory [15], antiviral [16], anticancer [17], antioxidant [18] and analgesic [19] activities.

Recent research in drug discovery aimed to introduce the 1,2,3-triazole moiety as a connecting unit to link together two or more pharmacophores for the design of novel bioactive molecules. Thus, it was hypothesized that the chemical combination of 1,2,4-triazole, 1,2,3-triazole and surfactants side chain in one scaffold may prove to be a breakthrough for chemical and biological activity as continuation of our effort in the designing of novel polyheterocyclic bioactive molecules [20–24].

In modern drug designing, molecular docking is routinely used for understanding drug- receptor interaction. Molecular docking provides useful information about drug receptor interactions and is frequently used to predict the binding orientation of small molecule drug candidates to their protein targets in order to predict the affinity and activity of the small molecule [25]. When designing novel antimicrobial agents, enzymes involved in the biosynthesis of microbial cell walls are generally good targets. In this regard, the enzyme glucosamine-6-phosphate synthase (GlmS, GlcN-6-P synthase, l-glutamine: d-fructose-6P amido-transferase, EC 2.6.1.16) is particularly attractive [26]. It is involved in the first step of the formation of the core amino-sugar, N-acetyl Glucosamine which is an essential building block of bacterial and fungal cell walls [27, 28]. Accordingly, GlcN-6-P serves as a promising target for antibacterial and antifungal drug discovery. Structural differences between prokaryotic and human enzymes may be exploited to design specific inhibitors, which may serve as prototypes of anti-fungal and anti-bacterial drugs [28]. Triazole type units have been reported to be good inhibitors of GlcN-6-P synthase [29–31]. Moreover, ciprofloxacin, the standard drug used for in vitro screenings in our studies, has been reported to be a good inhibitor of GlcN-6-P synthase [31–34]. Therefore, it was thought worthwhile to select GlcN-6-P synthase as the target for the synthesized triazole compounds.

## Results and discussion

### Chemistry

**Scheme 1:**
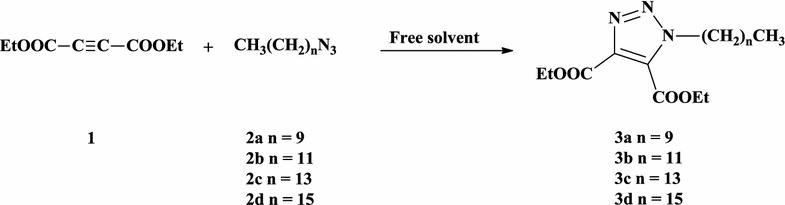
Synthesis of non-ionic surfactants based 1,2,3-triazole-4,5-diesters **3a**–**d**

An optimized eco-friendly click procedure has been previously developed in our laboratory for the construction of a series of novel 4,5-disubstituted 1,2,3-triazoles via 1,3-dipolar cycloaddition of dimethylacetylene dicarboxylate with different aromatic azides under solvent-free conditions. In the present work, we have investigated the applicability of the solvent-free conditions as a green procedure for the synthesis of novel non-ionic surfactants carrying 1,2,3-triazole and 1,2,4-triazole moieties. Thus, 1,3-dipolar cycloaddition of diethylacetylene dicarboxylate (**1**) with different surfactant azides **2a**–**d** under solvent free conditions, furnished the targeted non-ionic surfactants based 1,2,3-triazole-4,5-disesters **3a**–**d** in 95–98% yields (Scheme 1). The reaction required heating in a water bath for 3 min.

**Scheme 2:**
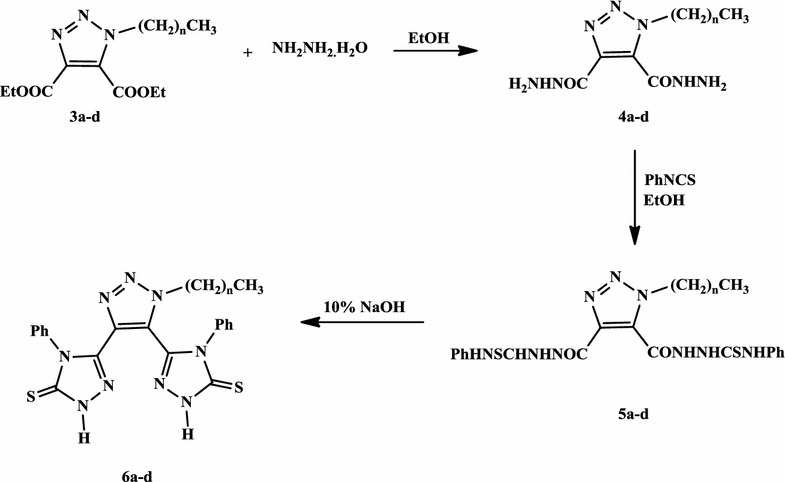
Synthesis of 1,2,3-triazole bis-1,2,4-triazole-3-thiones **6a**–**d**

The diacid hydrazides **4a**–**d** have been prepared successfully by stirring an ethanolic solution of the synthesized di-esters **3a**–**d** with hydrazine hydrate for 4 h at room temperature (Scheme 2). Thus, the condensation of the diacid hydrazides **4a**–**d** with phenyl isothiocyanate, in refluxing ethanol for 6 h, furnished the targeted phenylthiosemicarbazide derivatives **5a**–**d** in good yields (82–87%) (Scheme 2).

The 1,2,3-triazoles carrying bis-1,2,4-triazoles-3-thiones **6a**–**d** have been synthesized via intramolecular dehydrative ring closure of their corresponding thiosemicarbazide derivatives **5a**–**d** in 10% aqueous sodium hydroxide as basic catalyst as shown in Scheme 2. The reaction required heating under reflux for 6 h to afford compounds **6a**–**d** in good yields (80–85%).

**Scheme 3:**
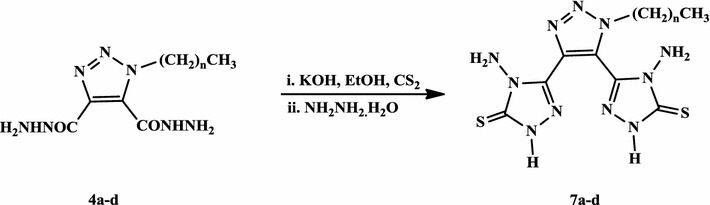
Synthesis of 1,2,3-triazole bis-4-amino-1,2,4-triazole-3-thiones **7a**–**d**

The synthesis of 4-amino-1,2,4-triazole-3-thione derivatives **7a**–**d** pass first through the formation of the appropriate potassium dithiocarbazinate salt through the reaction of the acid hydrazides **4a**–**d** with carbon disulphide in ethanolic potassium hydroxide solution (Scheme 3). The resulting potassium salts were then subjected to intramolecular ring closure, in the presence of hydrazine hydrate under reflux for 6 h, to afford 80–84% yields of the desired 4-amino-1,2,4-triazole-3-thiones **7a**–**d**.

The newly synthesized compounds were fully characterized based on their IR, ^1^H NMR and ^13^C NMR spectra. The IR spectra of the 1,2,3-triazole di-esters **3a**–**d** revealed the presence of strong absorption bands at 1738–1745 cm^−1^ assigned to the ester C=O groups. The ^1^H NMR spectrum of compound **3c** showed a quartet at δ_H_ 4.27–4.32 ppm and a multiplet at δ_H_ 4.40–4.48 ppm characteristic for the two non-equivalent ester methylene groups. The two ester methyl protons were recorded as a triplet integrated for six protons at δ_H_ 1.41 ppm. The proton spectral analysis also showed the surfactant proton signals on their appropriate aliphatic region (see “Experimental”). Its ^13^C NMR spectrum revealed no signals on the sp-carbon regions confirming the success of the cycloaddition reaction, and two characteristic signals appeared at δ_C_ 158.72 and 160.33 ppm attributed to the two ester carbonyl carbons (C=O). The surfactant side chain carbons appeared in their expected aliphatic region.

The success of the hydrazinolysis reaction was confirmed by the spectral data analysis of the diacid hydrazides **4a**–**d**. Their IR spectra showed characteristic NH and NH_2_ bands of the hydrazide functionalities near 3246–3367 cm^−1^. The ^1^H NMR spectrum of the diacid hydrazide **4b** was taken as example to confirm the success of the reaction. It showed the disappearance of the ethyl ester protons (CH_2_CH_3_) and the appearance of new multiplet at δ_H_ 4.74–4.79 ppm assignable to the NH_2_ and NCH_2_ groups. The two non-equivalent NH amide protons were assigned to two singlets at δ_H_ 10.42 and 11.83 ppm. The ^13^C NMR spectrum also confirmed the success of the hydrazinolysis reaction through, first the absence of the two ethoxy signals from their chemical shift regions, second the appearance of the two carbonyl hydrazide moieties at lower frequencies (δ_C_ 155.46 and 159.23 ppm) compared to their ester precursors (δ_C_ 158.72 and 160.33 ppm).

The IR spectra of the thiosemicarbazides **5a**–**d** revealed the presence of the thiocarbonyl groups (C=S) by the appearance of new absorption bands at 1289–1298 cm^−1^. The ^1^H NMR spectrum of compound **5a** was characterized by the disappearance of the NH_2_ signals and appearance of ten aromatic protons of the two phenyl rings at δ_H_ 7.12–7.74 ppm, confirmed the success of the condensation reaction. The two NH-protons bonded to the two phenyl groups appeared as two singlets at δ_H_ 9.64 and 9.67 ppm. The ^1^H NMR also showed four singlets at δ_H_ 9.90, 10.08, 11.23 and 11.55 ppm integrated for four protons related to the NH amidic (NHCO) and NH thioamidic (NHCS) protons of the two thiosemicarbazide moieties. The ^13^C NMR spectrum also approve the formation of the expected thiosemicarbazide product **5a** through the appearance of the aromatic carbons at δ_C_ 124.04–138.90 ppm and the presence of two characteristic signals at δ_C_ 180.18 and 181.07 ppm attributed to the two thiocarbonyl groups (C=S). Additionally, the spectrum revealed the aliphatic carbons for the surfactant side chain on their expected chemical shifts.

In the IR spectra of compounds **6a**–**d**, the absence of the carbonyl (C=O) and thiocarbonyl (C=S) absorption bands and the presence of new absorption band near 1608–1615 cm^−1^ characteristic for the C=N groups confirmed the success of the intramolecular ring closure to form 1,2,4-triazole-3-thione. In addition, the exhibited chemical shifts obtained from their ^1^H NMR, ^13^C NMR and spectra were all supported the proposed structures of **6a**–**d**. The ^1^H NMR spectrum of compound **6d** revealed the appearance of a diagnostic broad singlet at δ_C_ 10.60 ppm assignable to the NH’s of the thione isomer. The phenyl protons resonated as a multiplet at δ_H_ 7.02–7.49 ppm. In the ^13^C NMR spectrum of compound **6d**, the C=S signals appeared at 187.84 ppm confirming the predominance of the thione isomer. Furthermore, the aromatic carbons and the surfactant side chain carbons were observed on their appropriate chemical shifts.

The structures of the aminotriazoles **7a**–**d** have been also deduced from their elemental and spectral data. In their IR spectra, the presence of strong absorption bands at 1288–1296 and 3275–3380 cm^−1^ attributed to the C=S, NH and NH_2_ functional groups confirmed the formation of the 1,2,4-triazole ring. The ^1^H-NMR analysis revealed the presence of two diagnostic singlets at δ_H_ 5.19–5.27 ppm (NH_2_) and 9.21–9.31 ppm (NH), confirming the presence of the triazole ring in its thione form. In their ^13^C-NMR spectra, the presence of signals at δ_C_ 187.60–187.68 ppm attributed to the thiocarbonyl carbons (C=S), which were not observed on their corresponding starting hydrazides **4a**–**d** is another support for the predominance of the thione form.

### Antimicrobial evaluation

Antimicrobial activities of the newly synthesized compounds were evaluated against a panel of pathogenic microorganisms including Gram-positive bacteria, Gram-negative bacteria, and fungi. Antimicrobial activities were expressed as the Minimum Inhibitory Concentration (MIC) that is defined as the least concentration of the examined compound resulted in more than 80% growth inhibition of the microorganism [35, 36]. *Bacillus cereus, Enterococcus faecalis* and *Staphylococcus aureus* were used as model microorganisms representing Gram positive bacteria while *Proteus mirabilis, Escherichia coli* and *Pseudomonas aeruginosa* were used as representative of the Gram negative bacteria. On the other hand, *Candida albicans* and *Aspergillus brasiliensis* were chosen to study the antifungal activities of the synthesized compounds under examination (Table 1).

**Table 1:** Antimicrobial screening results of compounds **3**–**7(a**–**d)** expressed as MIC defined as the least concentration that cause more than 80% growth inhibition of the microorganism (μg/mL)

Compound no.	Gram-positive organisms			Gram-negative organisms			Fungi	
	*Bc*	*Ef*	*Sa*	*Pa*	*Ec*	*Pm*	*Ab*	*Ca*
**3a**	256	512	512	128	256	512	512	256
**3b**	128	512	256	128	128	512	512	256
**3c**	64	256	128	64	128	256	256	128
**3d**	64	256	128	64	64	256	256	128
**4a**	128	256	128	64	128	256	256	128
**4b**	64	256	64	64	128	256	256	128
**4c**	64	128	64	32	64	128	64	64
**4d**	32	128	64	16	32	128	64	32
**5a**	32	128	64	32	64	256	128	64
**5b**	32	128	32	32	32	128	128	32
**5c**	16	64	32	16	32	64	32	16
**5d**	8	64	16	8	16	64	32	16
**6a**	16	64	32	16	16	128	32	8
**6b**	16	64	16	16	16	64	32	8
**6c**	4	32	8	8	8	32	16	4
**6d**	4	32	4	4	4	32	16	2
**7a**	8	32	16	8	8	64	16	4
**7b**	8	16	8	8	8	64	16	4
**7c**	2	16	4	4	4	32	8	2
**7d**	2	8	1	2	1	16	8	1
Ciprofloxacin	4	8	1	4	1	8	–	–
Fluconazole	–	–	–	–	–	–	4	1

*Bacillus cereus* ATTC 10876 (*B. cereus*), *Enterococcus faecalis* ATTC 29212 (*E.* *faecalis), Staphylococcus aureus* ATTC 25923 (*S. aureus*)

*Proteus mirabilis* ATTC 35659 (*P. mirabilis*), *Escherichia coli* ATTC 25922 (*E. coli*), *Pseudomonas aeruginosa* ATTC 27853 (*P. aeruginosa*)

*Candida albicans* ATTC 50193 (*C. albicans*), *Aspergillus brasiliensis* ATTC 16404 (*A. brasiliensis*)

*MIC* minimum inhibitory concentration

Antibacterial and antifungal screening revealed that some of the examined compounds demonstrated fair to excellent antimicrobial activities relative to Ciprofloxacin and Fluconazole; standard potent antibacterial and antifungal, respectively. Among the studied compounds, **7a**–**d** emerged as the most potent antimicrobial agents relative to the standards, with MIC ranges between 1 and 32 µg/mL against Gram positive bacteria, 1–64 µg/mL against Gram negative bacteria and 1–16 µg/mL against fungi. Compared to Ciprofloxacin, compound 5,5′-(1-hexadecyl-1*H*-1,2,3-triazole-4,5-diyl)bis(4-amino-1,2,4-triazole-5(4*H*)-thione) (**7d**) appears to exert similar or more potent antibacterial activities against all bacterial species tested. Likewise, compound **7d** demonstrates a comparable antifungal activity to that of the potent standard Fluconazole. Interestingly, increasing the carbon chain length substitution on the 1,2,3-triazole moiety of the 1,2,3-triazole-bis-4-amino-1,2,4-triazole-3-thiones **7a**–**d** resulted in 2–16-folds improvement of the antimicrobial activity.

Interestingly, 1,2,3-triazole-4,5-diyl)bis(4-phenyl-2,4-dihydro-1,2,4-triazole-3-thione derivatives **6a**–**d** revealed similar trend of activity to that associated with the 1,2,3-triazole bis-4-amino-1,2,4-triazole-3-thione derivatives **7a**–**d** indicating an improved antimicrobial activity of the 1,2,4 triazole moiety. MIC ranges between 4 and 64 µg/mL against Gram positive bacteria, 4–128 µg/mL against Gram negative bacteria, and 2–64 µg/mL against fungi. Nonetheless, 1,2,3-triazole derivatives with the triazole bis-4-amino-1,2,4-triazole-3-thiones substitution **7a**–**d** appears to have superior antimicrobial activities over the 1,2,3-triazole-4,5-diyl)bis (4-phenyl-2,4-dihydro-1,2,4-triazole-3-thione derivatives **6a**–**d** suggesting a balanced hydrophylicity/hydrophobicity ratio that results in a better penetration though microorganisms’ cellular membranes; hence, augmented activities. Similarly, increasing carbon chain length of the 1,2,3-triazole moiety enhanced the effectiveness of the 1,2,3-triazole-bis-1,2,4-triazole-3-thione derivatives **6a**–**d**.

On the other hand, 1,2,3-triazole bis-acid thiosemicarbazide derivatives **5a**–**d** yielded intermediate antibacterial and antifungal activities relative to both standards, Ciprofloxacin and Fluconazole. MIC ranges between 8 and 128 µg/mL against Gram positive bacteria, 8–256 µg/mL against Gram negative bacteria, and 16–128 µg/mL against fungi. The diminished activity is probably due to the loss of the 1,2,4-triazole moiety. Structural activity relationship suggests that extending the N-1 alkyl substitution from the decyl to hexadecyl chain will enhance the antimicrobial activity by fourfolds. Whereas 1-hexadecyl-1,2,3-triazole-4,5-diyl)-bis(4-*N*-phenylacid thiosemicarbazide (**5d**) demonstrates a promising activity, relative to **5a**, **5b**, and **5c**, against the examined strains, it is still less efficient as antimicrobial than the 1,2,4-triazole derivatives.

In view of that, 1,2,3-triazole-4,5-diesters **3a**–**d** and 1,2,3-triazole diacid hydrazides **4a**–**d** were evidently less efficient to exert comparable antimicrobial activities to the previously observed activities associated with the substituted 1,2,4-triazole derivatives. Remarkably, 1,2,3-triazole-4,5-diesters **3a**–**d** exhibited the least efficient antimicrobial activities against all microorganisms with MIC values ranging from 64 to 512 µg/mL against Gram positive bacteria and Gram negative bacteria, and 128–512 µg/mL against fungi. Diethyl-(1-decyl-1,2,3-triazole-4,5-diyl)diformate (**3a**) appears to have the least potency as an antifungal agent relative to Fluconazole. Chain extension of the N-1 alkyl substitution yielded twofolds enhancement in the antifungal activity and two to fourfolds enhancement in the antibacterial activity.

1,2,3-Triazole diacid hydrazide derivatives **4a**–**d** show a better activity than 1,2,3-triazole-4,5-diesters **3a**–**d** with MIC ranging from 32 to 256 µg/mL against Gram positive bacteria, 16–256 µg/mL against Gram negative bacteria, and 32–256 µg/mL against fungi. Analogously, increasing the hydrophobicity at the *N*-1 position of the 1,2,3-triazole will most likely facilitate a better cellular membrane penetration and consequently an enhanced antimicrobial activity.

Consistent with previous reports [20], and on the basis of the observed MIC values for the examined compounds, it was concluded that 1,2,4-triazole derivatives with elongated chain substitution at the 1,2,3-triazole *N*-1 position likely exhibit enhanced antibacterial and antifungal activities over analogous 1,2,4-triazole derivatives.

### In-silico screenings (molecular docking)

**Fig. 1:**
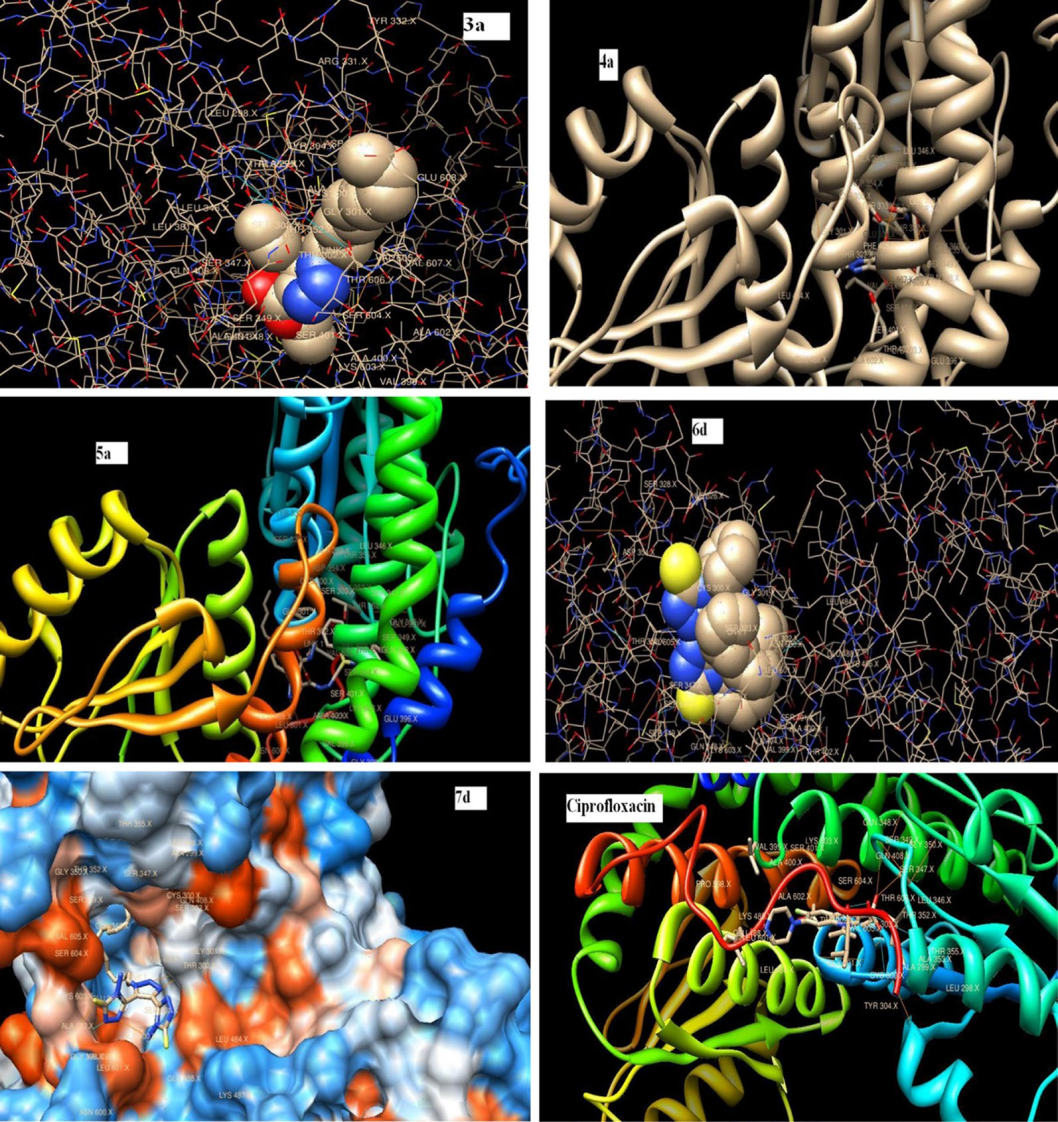
Docking of some compounds **3a**, **4a**, **5a**, **6d**, **7d** and standard drug ciprofloxacin into active site of glucosamine-6-phosphate (GlcN-6-P) synthase

In correlation to in vitro antimicrobial activity, it was thought worthy to perform molecular docking studies, hence screening the compounds, inculcating both in silico and in vitro results. The amino sugars are the significant building blocks of polysaccharides found in the cell wall of most human pathogenic microorganisms. Therefore not surprising that a number of GlcN-6-P synthase inhibitors of natural or synthetic origin display bactericidal or fungicidal properties [37]. Considering GlcN-6-P synthase as the target receptor, comparative and automated docking studies with newly synthesized candidate lead compounds was performed to determine the best in silico conformation. The molecular docking of the synthesized compounds with GlcN-6-P synthase revealed that all tested compounds have shown the bonding with one or the other amino acids in the active pockets. Figure 1 shows the docked images of selected candidate ligands including the considered standard drug i.e. Ciprofloxacin. Table 2 shows the binding energy and inhibition constant of the tested compounds including the standard. In-silico studies revealed all the synthesized molecules showed good binding energy toward the target protein ranging from − 5.72 to − 10.49 kJ mol^−1^.

**Table 2:** Molecular docking results of the target compounds

Compound no.	Minimum binding energy (kcal/mol)	Estimated inhibition constant, Ki = μM (micromolar), nM (nanomolar)
**3a**	− 6.35	21.99 μM
**3b**	− 5.72	63.71 μM
**3c**	− 6.85	9.46 μM
**3d**	− 6.61	14.20 μM
**4a**	− 8.03	1.30 μM
**4b**	− 7.67	2.40 μM
**4c**	− 7.55	2.94 μM
**4d**	− 6.88	9.04 μM
**5a**	− 9.31	150.86 nM
**5b**	− 7.92	1.57 μM
**5c**	− 6.33	22.73 μM
**5d**	− 6.27	25.31 μM
**6a**	− 9.24	167.77 nM
**6b**	− 9.77	69.47 nM
**6c**	− 10.33	26.60 nM
**6d**	− 10.49	20.57 nM
**7a**	− 8.86	320.85 nM
**7b**	− 9.27	159.60 nM
**7c**	− 9.30	151.56 nM
**7d**	− 9.23	170.12 nM
Ciprofloxacin	− 6.28	24.97 μM

## Experimental

### General chemistry

Melting points were recorded on a Stuart Scientific SMP1 apparatus and are uncorrected. The IR spectra were measured using an FTIR-8400 s-Fourier transform infrared spectrophotometer-Shimadzu. The NMR spectra were determined on Advance Bruker NMR spectrometer at 400 MHz with TMS as internal standard. The ESI mass spectra were measured by a Finnigan LCQ spectrometer.

#### Synthesis and characterization of 1,2,3-triazole di-esters **3a**–**d**

Diethyl acetylenedicarboxylate **1** (15 mmol) and the appropriate surfactant azide **2a**–**d** (20 mmol) were heated on a water bath for 3 min. The reaction mixture was cooled and then ether was added to precipitate the product. The solid was filtered and washed with hexane.

##### Characterization of diethyl 1-decyl-1*H*-1,2,3-triazole-4,5-dicarboxylate (**3a**)

It was obtained in 98% (hygroscopic). IR (KBr): 1742 (C=O), 1572 (C=C) cm^−1^. ^1^H NMR (400 MHz, CDCl_3_): δ_H_ = 0.86 (t, 3H, *J* = 8 Hz, C**H**_3_), 1.22–1.27 (m, 14H, 7 × C**H**_2_), 1.40 (t, 6H, *J* = 8 Hz, 2 × OCH_2_C**H**_3_), 1.77–1.82 (m, 2H, NCH_2_C**H**_2_), 3.37 (dd, 1H, *J* = 4 Hz, 8 Hz, NC**H**_2_), 4.23–4.30 (q, 1H, *J* = 4 Hz, 8 Hz, OC**H**_2_CH_3_), 4.41–4.47 (m, 3H, OC**H**_2_CH_3_), 4.70 (t, 1H, *J* = 8 Hz, NC**H**_2_). ^13^C NMR (100 MHz, CDCl_3_): δ_C_ = 13.95 (**C**H_3_), 14.12, 14.22 (OCH_2_ **C**H_3_), 22.84, 26.54, 28.30, 28.79, 29.24, 29.63, 29.84, 29.99, 30.54, 32.71, 33.65 (**C**H_2_), 50.97 (N**C**H_2_), 61.80, 62.87 (2 × O**C**H_2_CH_3_), 129.46, 140.14, 151.98, 158.35, 160.87 (**C**=C, **C**=O). Anal. Calcd. for C_18_H_31_N_3_O_4_: C, 61.17; H, 8.84; N, 11.89. Found: C, 61.29; H, 8.79; N, 11.80. ESI MS (*m/z*): 354.23 [M+H]^+^.

##### Characterization of diethyl 1-dodecyl-1*H*-1,2,3-triazole-4,5-dicarboxylate (**3b**)

It was obtained in 97% (hygroscopic). IR (KBr): 1745 (C=O), 1566 (C=C) cm^−1^. ^1^H NMR (400 MHz, CDCl_3_): δ_H_ = 0.85 (t, 3H, *J* = 8 Hz, C**H**_3_), 1.20–1.26 (m, 18H, 9 × C**H**_2_), 1.43 (t, 6H, *J* = 8 Hz, 2 × OCH_2_C**H**_3_), 1.75–1.80 (m, 2H, NCH_2_C**H**_2_), 3.44 (dd, 1H, *J* = 4 Hz, 8 Hz, NC**H**_2_), 4.20–4.28 (q, 1H, *J* = 4 Hz, 8 Hz, OC**H**_2_CH_3_), 4.35–4.42 (m, 3H, OC**H**_2_CH_3_), 4.71 (t, 1H, *J* = 8 Hz, NC**H**_2_). ^13^C NMR (100 MHz, CDCl_3_): δ_C_ = 13.90 (**C**H_3_), 14.19, 14.28 (OCH_2_ **C**H_3_), 22.80, 26.59, 26.77, 28.46, 28.80, 29.07, 29.26, 29.80, 29.92, 30.22, 30.64, 32.83, 33.83 (**C**H_2_), 50.85 (N**C**H_2_), 61.73, 62.65 (2 × O**C**H_2_CH_3_), 129.44, 140.28, 151.83, 158.40, 160.95 (**C**=C, **C**=O). Anal. Calcd. for C_20_H_35_N_3_O_4_: C, 62.96; H, 9.25; N, 11.01. Found: C, 62.88; H, 9.32; N, 11.12. ESI MS (*m/z*): 382.26 [M+H]^+^.

##### Characterization of diethyl 1-tetradecyl-1*H*-1,2,3-triazole-4,5-dicarboxylate (**3c**)

It was obtained in 96% (hygroscopic). IR (KBr): 1738 (C=O), 1580 (C=C) cm^−1^. ^1^H NMR (400 MHz, CDCl_3_): δ_H_ = 0.88 (t, 3H, *J* = 8 Hz, C**H**_3_), 1.26–1.33 (m, 22H, 11 × C**H**_2_), 1.41 (t, 6H, *J* = 8 Hz, 2 × OCH_2_C**H**_3_), 1.81–1.91 (m, 2H, NCH_2_C**H**_2_), 3.41 (dd, 1H, *J* = 4 Hz, 8 Hz, NC**H**_2_), 4.27–4.32 (q, 1H, *J* = 4 Hz, 8 Hz, OC**H**_2_CH_3_), 4.40–4.48 (m, 3H, OC**H**_2_CH_3_), 4.58 (t, 1H, *J* = 8 Hz, NC**H**_2_). ^13^C NMR (100 MHz, CDCl_3_): δ_C_ = 13.95 (**C**H_3_), 14.12, 14.22 (OCH_2_ **C**H_3_), 22.73, 26.39, 28.24, 28.38, 28.99, 29.40, 29.50, 29.53, 29.60, 29.64, 29.68, 30.29, 31.97, 32.91, 33.90 (**C**H_2_), 50.55 (N**C**H_2_), 61.78, 62.98 (2 × O**C**H_2_CH_3_), 129.97, 140.22, 151.79, 158.72, 160.33 (**C**=C, **C**=O). Anal. Calcd. For C_22_H_39_N_3_O_4_: C, 64.52; H, 9.60; N, 10.26; Found: C, 64.71; H, 9.52; N, 10.18. ESI MS (*m/z*): 410.29 [M+H]^+^.

##### Characterization of diethyl 1-hexadecyl-1*H*-1,2,3-triazole-4,5-dicarboxylate (**3d**)

It was obtained in 95% (hygroscopic). IR (KBr): 1740 (C=O), 1575 (C=C) cm^−1^. ^1^H NMR (400 MHz, CDCl_3_): δ_H_ = 0.85 (t, 3H, *J* = 8 Hz, C**H**_3_), 1.23–1.34 (m, 26H, 13 × C**H**_2_), 1.49 (t, 6H, *J* = 8 Hz, 2 × OCH_2_C**H**_3_), 1.84–1.90 (m, 2H, NCH_2_C**H**_2_), 3.50 (dd, 1H, *J* = 4 Hz, 8 Hz, NC**H**_2_), 4.23–4.30 (q, 1H, *J* = 4 Hz, 8 Hz, OC**H**_2_CH_3_), 4.37–4.45 (m, 3H, OC**H**_2_CH_3_), 4.52 (t, 1H, *J* = 8 Hz, NC**H**_2_). ^13^C NMR (100 MHz, CDCl_3_): δ_C_ = 13.87 (**C**H_3_), 14.23, 14.28 (OCH_2_ **C**H_3_), 22.70, 26.34, 28.29, 28.54, 28.90, 29.45, 29.59, 29.87, 29.99, 30.11, 30.43, 30.64, 31.66, 32.45, 33.56, 33.87 (**C**H_2_), 50.47 (N**C**H_2_), 61.86, 62.73 (2 × O**C**H_2_CH_3_), 129.92, 140.85, 152.33, 158.80, 161.24 (**C**=C, **C**=O). Anal. Calcd. For C_24_H_43_N_3_O_4_: C, 65.87; H, 9.90; N, 9.60. Found: C, 65.94; H, 9.82; N, 9.72. ESI MS (*m/z*): 438.32 [M+H]^+^.

#### Synthesis and characterization of 1,2,3-triazole di-acid hydrazides **4a**–**d**

A mixture of compound **3a**–**d** (10 mmol) and hydrazine hydrate (20 mmol) in ethanol (50 mL) was stirred for 5–15 min at rt. Ethanol was removed under reduced pressure, and the product formed was recrystallized from ethanol to give the titled compounds **4a**–**d**.

##### Characterization of 1-decyl-1*H*-1,2,3-triazole-4,5-dicarbohydrazide (**4a**)

It was obtained in 91% as colorless crystals, mp: 125–126 °C. IR (KBr): 3273–3367 (NH, NH_2_), 1690 (C=O), 1565 (C=C) cm^−1^. ^1^H NMR (400 MHz, DMSO-*d*_*6*_): δ_H_ = 0.85 (t, 3H, *J* = 8 Hz, C**H**_3_), 1.23 (bs, 14H, 7 × C**H**_2_), 1.78–1.82 (m, 2H, NCH_2_C**H**_2_), 4.73–4.78 (m, 6H, NC**H**_2_, 2 × N**H**_2_), 10.42 (s, 1H, N**H**), 11.84 (s, 1H, N**H**). ^13^C NMR (100 MHz, DMSO-*d*_*6*_): δ_C_ = 13.90 (**C**H_3_), 22.06, 25.76, 28.36, 28.62, 28.82, 29.80, 31.23 (**C**H_2_), 50.32 (N**C**H_2_), 129.42, 137.82, 155.46, 159.22 (**C**=C, **C**=O). Anal. Calcd. For C_14_H_27_N_7_O_2_: C, 51.67; H, 8.36; N, 30.13. Found: C, 51.81; H, 8.32; N, 30.21. ESI MS (*m/z*): 326.22 [M+H]^+.^

##### Characterization of 1-dodecyl-1*H*-1,2,3-triazole-4,5-dicarbohydrazide (**4b**)

It was obtained in 90% as colorless crystals, mp: 115–116 °C. IR (KBr): 3254–3365 (NH, NH_2_), 1694 (C=O), 1579 (C=C) cm^−1^. ^1^H NMR (400 MHz, DMSO-*d*_*6*_): δ_H_ = 0.85 (t, 3H, *J* = 8 Hz, C**H**_3_), 1.23 (bs, 18H, 9 × C**H**_2_), 1.78–1.81 (m, 2H, NCH_2_C**H**_2_), 4.74–4.79 (m, 2H, NC**H**_2_, 2 × N**H**_2_), 10.42 (s, 1H, N**H**), 11.83 (s, 1H, N**H**). ^13^C NMR (100 MHz, DMSO-*d*_*6*_): δ_C_ = 13.91 (**C**H_3_), 22.06, 25.77, 28.37, 28.67, 28.82, 28.89, 28.96, 28.97, 29.81, 31.25 (**C**H_2_), 50.32 (N**C**H_2_), 129.43, 137.82, 155.46, 159.23 (**C**=C, **C**=O). Anal. Calcd. For C_16_H_31_N_7_O_2_: C, 54.37; H, 8.84; N, 27.74. Found: C, 54.41; H, 8.74; N, 27.80. ESI MS (*m/z*): 354.25 [M+H]^+^.

##### Characterization of 1-tetradecyl-1*H*-1,2,3-triazole-4,5-dicarbohydrazide (**4c**)

It was obtained in 88% as colorless crystals, mp: 110–111 °C. IR (KBr): 3267–3356 (NH, NH_2_), 1686 (C=O), 1569 (C=C) cm^−1^. ^1^H NMR (400 MHz, CDCl_3_): δ_H_ = 0.89 (t, 3H, *J* = 8 Hz, C**H**_3_), 1.26–1.35 (m, 22H, 11 × C**H**_2_), 1.88–1.96 (m, 2H, NCH_2_C**H**_2_), 4.19 (bs, 4H, 2 × N**H**_2_), 4.93 (dd, 2H, *J* = 4 Hz, 8 Hz, NC**H**_2_), 7.28 (s, 1H, N**H**), 12.06 (s, 1H, N**H**). ^13^C NMR (100 MHz, CDCl_3_): δ_C_ = 14.06 (**C**H_3_), 22.64, 26.47, 29.02, 29.31, 29.41, 29.49, 29.57, 29.61, 29.64, 30.52, 31.88 (**C**H_2_), 51.80 (N**C**H_2_), 129.36, 137.31, 156.73, 161.87 (**C**=C, **C**=O). Anal. Calcd. For C_18_H_35_N_7_O_2_: C, 56.67; H, 9.25; N, 25.70. Found: C, 56.80; H, 9.30; N, 25.77. ESI MS (*m/z*): 382.28 [M+H]^+^.

##### Characterization of 1-hexadecyl-1*H*-1,2,3-triazole-4,5-dicarbohydrazide (**4d**)

It was obtained in 85% as colorless crystals, mp: 103–104 °C. IR (KBr): 3246–3361 (NH, NH_2_), 1697 (C=O), 1575 (C=C) cm^−1^. ^1^H NMR (400 MHz, CDCl_3_): δ_H_ = 0.87 (t, 3H, *J* = 8 Hz, C**H**_3_), 1.25–1.37 (m, 26H, 13 × C**H**_2_), 1.86–1.92 (m, 2H, NCH_2_C**H**_2_), 4.21 (bs, 4H, 2 × N**H**_2_), 4.90 (dd, 2H, *J* = 4 Hz, 8 Hz, NC**H**_2_), 7.24 (s, 1H, N**H**), 12.11 (s, 1H, N**H**). ^13^C NMR (100 MHz, CDCl_3_): δ_C_ = 14.09 (**C**H_3_), 22.69, 26.73, 29.23, 29.57, 29.70, 29.98, 30.34, 30.46, 30.59, 30.72, 31.64, 31.93 (**C**H_2_), 51.76 (N**C**H_2_), 129.56, 137.49, 156.97, 159.55 (**C**=C, **C**=O). Anal. Calcd. For C_20_H_39_N_7_O_2_: C, 58.65; H, 9.60; N, 23.94. Found: C, 58.74; H, 9.66; N, 23.89. ESI MS (*m/z*): 410.31 [M+H]^+^.

#### Synthesis and characterization of 1,2,3-triazole bis-acid thiosemicarbazides **5a**–**d**

A mixture of compound **4a**–**d** (10 mmol) and phenyl isothiocyanate (20 mmol) in ethanol (50 ml) was refluxed for 6 h. The solution was cooled and a white solid appeared. The obtained precipitate was filtered and recrystallized from ethanol to give the titled compounds **5a**–**d**.

##### Characterization of 2,2′-(1-decyl-1*H*-1,2,3-triazole-4,5-dicarbonyl)bis(*N*-phenylhydrazine-carbothioamide) (**5a**)

It was obtained in 87% as colorless crystals, mp: 187–188 °C. IR (KBr): 3237–3377 (NH), 1694 (C=O), 1570 (C=C), 1298 (C=S) cm^−1^. ^1^H NMR (400 MHz, DMSO-*d*_*6*_): δ_H_ = 0.85 (t, 3H, *J* = 8 Hz, C**H**_3_), 1.24–1.27 (m, 14H, 7 × C**H**_2_), 1.83–1.86 (m, 2H, NCH_2_C**H**_2_), 4.60 (bs, 2H, NC**H**_2_), 7.12–7.17 (m, 2H, Ar–**H**), 7.27–7.33 (m, 6H, Ar–**H**), 7.69–7.74 (m, 2H, Ar–**H**), 9.64, 9.67 (2bs, 2H, 2 × N**H**Ph), 9.90, 10.08 (2 s, 2H, 2 × N**H**CS), 11.23, 11.55 (2bs, 2H, 2 × CON**H**). ^13^C NMR (100 MHz, DMSO-*d*_*6*_): δ_C_ = 13.86 (**C**H_3_), 21.99, 25.72, 28.29, 28.57, 28.77, 28.84, 29.52, 31.18 (**C**H_2_), 49.73 (N**C**H_2_), 124.04, 124.77, 125.17, 126.06, 128.06, 131.14, 138.66, 138.90 (Ar–**C**), 157.30, 160.52, 180.18, 181.07 (**C**=O, **C**=S). Anal. Calcd. For C_28_H_37_N_9_O_2_S_2_: C, 56.45; H, 6.26; N, 21.16. Found: C, 56.36; H, 6.18; N, 21.05. ESI MS (*m/z*): 596.25 [M+H]^+^.

##### Characterization of 2,2′-(1-dodecyl-1*H*-1,2,3-triazole-4,5-dicarbonyl)bis(*N*-phenylhydrazine-carbothioamide (**5b**)

It was obtained in 86% as colorless crystals, mp: 180–181 °C. IR (KBr): 3248–3360 (NH), 1698 (C=O), 1581 (C=C), 1295 (C=S) cm^−1^. ^1^H NMR (400 MHz, DMSO-*d*_*6*_): δ_H_ = 0.86 (t, 3H, *J* = 8 Hz, C**H**_3_), 1.24–1.27 (m, 18H, 9 × C**H**_2_), 1.81–1.87 (m, 2H, NCH_2_C**H**_2_), 4.62 (bs, 2H, NC**H**_2_), 7.10–7.19 (m, 2H, Ar–**H**), 7.23–7.30 (m, 6H, Ar–**H**),) 7.68–7.73 (m, 2H, Ar–**H**), 9.68, 9.88 (2bs, 2H, 2 × N**H**Ph), 9.67, 9.72 (2 s, 2H, 2 × N**H**CS), 11.20, 11.51 (2bs, 2H, 2 × CON**H**). ^13^C NMR (100 MHz, DMSO-*d*_*6*_): δ_C_ = 13.84 (**C**H_3_), 21.96, 25.70, 28.34, 28.63, 28.75, 28.88, 29.57, 29.77, 30.09, 31.28 (**C**H_2_), 49.79 (N**C**H_2_), 124.09, 124.80, 125.21, 126.11, 128.05, 131.19, 138.72, 138.95 (Ar–**C**), 157.36, 160.56, 180.29, 181.38 (**C**=O, **C**=S). Anal. Calcd. For C_30_H_41_N_9_O_2_S_2_: C, 57.76; H, 6.62; N, 20.21. Found: C, 57.66; H, 6.55; N, 20.16. ESI MS (*m/z*): 624.28 [M+H]^+^.

##### Characterization of 2,2′-(1-tetradecyl-1*H*-1,2,3-triazole-4,5-dicarbonyl)bis(*N*-phenylhydrazine-carbothioamide) (**5c**)

It was obtained in 82% as colorless crystals, mp: 173–174 °C. IR (KBr): 3255–3380 (NH), 1686 (C=O), 1580 (C=C), 1291 (C=S) cm^−1^. ^1^H NMR (400 MHz, DMSO-*d*_*6*_): δ_H_ = 0.86 (t, 3H, *J* = 8 Hz, C**H**_3_), 1.24–1.27 (m, 22H, 11 × C**H**_2_), 1.83–1.88 (m, 2H, NCH_2_C**H**_2_), 4.63 (bs, 2H, NC**H**_2_), 7.10–7.19 (m, 2H, Ar–**H**), 7.23–7.28 (m, 6H, Ar–**H**), 7.69–7.75 (m, 2H, Ar–**H**), 9.62, 9.65 (2bs, 2H, 2 × N**H**Ph), 9.93, 10.00 (2 s, 2H, 2 × N**H**CS), 11.28, 11.50 (2bs, 2H, 2 × CON**H**). ^13^C NMR (100 MHz, DMSO-*d*_*6*_): δ_C_ = 13.86 (**C**H_3_), 21.99, 25.72, 28.29, 28.57, 28.77, 28.84, 29.52, 31.18 (**C**H_2_), 49.73 (N**C**H_2_), 124.04, 124.77, 125.17, 126.06, 128.06, 131.14, 138.66, 138.90 (Ar–**C**), 157.30, 160.52, 180.18, 181.07 (**C**=O, **C**=S). Anal. Calcd. For C_32_H_45_N_9_O_2_S_2_: C, 58.96; H, 6.96; N, 19.34. Found: C, 58.85; H, 6.85; N, 19.41. ESI MS (*m/z*): 652.31 [M+H]^+^.

##### Characterization of 2,2′-(1-hexadecyl-1*H*-1,2,3-triazole-4,5-dicarbonyl)bis(*N*-phenylhydrazine-carbothioamide) (**5d**)

It was obtained in 85% as colorless crystals, mp: 160–161 °C. IR (KBr): 3252–3351 (NH), 1690 (C=O), 1574 (C=C), 1289 (C=S) cm^−1^. ^1^H NMR (400 MHz, DMSO-*d*_*6*_): δ_H_ = 0.87 (t, 3H, *J* = 8 Hz, C**H**_3_), 1.20–1.29 (m, 26H, 13 × C**H**_2_), 1.86–1.89 (m, 2H, NCH_2_C**H**_2_), 4.65 (bs, 2H, NC**H**_2_), 7.14–7.19 (m, 2H, Ar–**H**), 7.25–7.30 (m, 6H, Ar–**H**), 7.70–7.75 (m, 2H, Ar–**H**), 9.60, 9.64 (2bs, 2H, 2 × N**H**Ph), 9.88, 10.05 (2 s, 2H, 2 × N**H**CS), 11.24, 11.52 (2bs, 2H, 2 × CON**H**). ^13^C NMR (100 MHz, DMSO-*d*_*6*_): δ_C_ = 13.80 (**C**H_3_), 21.95, 25.75, 28.33, 28.59, 28.68, 28.79, 28.99, 29.44, 29.59, 31.24 (**C**H_2_), 49.64 (N**C**H_2_), 124.11, 124.80, 125.34, 126.12, 128.56, 131.49, 138.95, 139.06 (Ar–**C**), 157.43, 160.69, 180.76, 181.27 (**C**=O, **C**=S). Anal. Calcd. For C_34_H_49_N_9_O_2_S_2_: C, 60.06; H, 7.26; N, 18.54. Found: C, 60.13; H, 7.32; N, 18.47. ESI MS (*m/z*): 680.34 [M+H]^+^.

#### Synthesis and characterization of 1,2,3-triazole bis-1,2,4-triazole-3-thiones **6a**–**d**

A mixture of compound **5a**–**d** (10 mmol) and 10% aqueous sodium hydroxide solution (200 mL) was refluxed for 6 h. The mixture was then cooled to room temperature and filtered. The filtrate was acidified by the addition of hydrochloric acid. The resulting solid was collected by filtration, washed with water and recrystallized from ethanol to give compound **6a**–**d**.

##### Characterization of 5,5′-(1-decyl-1*H*-1,2,3-triazole-4,5-diyl)bis(4-phenyl-2,4-dihydro-1,2,4-triazole-3-thione) (**6a**)

It was obtained in 80% as colorless crystals, mp: 220–221 °C. IR (KBr): 3345 (NH), 1615 (C=N), 1570 (C=C), 1295 (C=S) cm^−1^. ^1^H-NMR (400 MHz, CDCl_3_): δ_H_ = 0.87–0.91 (m, 3H, C**H**_3_), 1.27–1.43 (m, 14H, 7 × C**H**_2_), 1.80–1.85 (m, 2H, NCH_2_C**H**_2_), 4.22–4.26 (m, 2H, NC**H**_2_), 7.10–7.46 (m, 10H, Ar–**H**), 9.08 (bs, 2H, 2 × **N**H). ^13^C NMR (100 MHz, CDCl_3_): δ_C_ = 14.10 (**C**H_3_), 15.21, 22.63, 26.22, 26.37, 28.85, 29.24, 29.31, 29.44, 29.93 (**C**H_2_), 31.83 (N**C**H_2_), 118.14, 121.72, 125.35, 127.78, 128.42, 128.97, 129.66, 137.31, 141.95, 188.58 (Ar–**C**, **C**=N, **C**=S). Anal. Calcd. For C_28_H_33_N_9_S_2_: C, 60.08; H, 5.94; N, 22.52. Found: C, 60.19; H, 5.85; N, 22.44. ESI MS (*m/z*): 560.23 [M+H]^+^.

##### Characterization of 5,5′-(1-dodecyl-1*H*-1,2,3-triazole-4,5-diyl)bis(4-phenyl-2,4-dihydro-1,2,4-triazole-3-thione) (**6b**)

It was obtained in 84% as colorless crystals, mp: 229–230 °C. IR (KBr): 3332 (NH), 1608 (C=N), 1578 (C=C), 1291 (C=S) cm^−1^. ^1^H-NMR (400 MHz, CDCl_3_): δ_H_ = 0.88 (t, 3H, *J* = 8 Hz, C**H**_3_), 1.28–1.45 (m, 18H, 9 × C**H**_2_), 1.81–1.88 (m, 2H, NCH_2_C**H**_2_), 4.20–4.28 (m, 2H, NC**H**_2_), 7.05–7.40 (m, 10H, Ar–**H**), 9.15 (bs, 2H, 2 × **N**H). ^13^C NMR (100 MHz, CDCl_3_): δ_C_ = 14.08 (**C**H_3_), 15.25, 22.78, 22.90, 26.31, 26.56, 28.80, 29.05, 29.29, 29.58, 29.73, 29.99, 30.23 (**C**H_2_), 31.97 (N**C**H_2_), 118.19, 121.46, 125.74, 127.69, 128.39, 128.87, 129.74, 137.47, 141.47, 188.70 (Ar–**C**, C=N, C=S). Anal. Calcd. For C_30_H_37_N_9_S_2_: C, 61.30; H, 6.34; N, 21.45. Found: C, 61.18; H, 6.43; N, 21.40. ESI MS (*m/z*): 588.26 [M+H]^+^.

##### Characterization of 5,5′-(1-tetradecyl-1*H*-1,2,3-triazole-4,5-diyl)bis(4-phenyl-2,4-dihydro-1,2,4-triazole-3-thione) (**6c**)

It was obtained in 83% as colorless crystals, mp: 238–239 °C. IR (KBr): 3365 (NH), 1611 (C=N), 1572 (C=C), 1297 (C=S) cm^−1^. ^1^H-NMR (400 MHz, CDCl_3_): δ_H_ = 0.87 (t, 3H, *J* = 8 Hz, C**H**_3_), 1.26–1.40 (m, 22H, 11 × C**H**_2_), 1.80–1.86 (m, 2H, NCH_2_C**H**_2_), 4.22–4.29 (m, 2H, NC**H**_2_), 7.09–7.43 (m, 10H, Ar–**H**), 9.12 (bs, 2H, 2 × **N**H). ^13^C NMR (100 MHz, CDCl_3_): δ_C_ = 14.14 (**C**H_3_), 15.26, 22.70, 22.96, 26.36, 26.54, 28.85, 29.09, 29.41, 29.72, 29.79, 29.94, 30.08, 30.38 (**C**H_2_), 31.88 (N**C**H_2_), 118.21, 121.51, 125.79, 127.72, 128.43, 128.84, 129.71, 137.45, 141.49, 188.59 (Ar–**C**, **C**=N, **C**=S). Anal. Calcd. For C_32_H_41_N_9_S_2_: C, 62.41; H, 6.71; N, 20.47. Found: C, 62.29; H, 6.65; N, 20.43. ESI MS (*m/z*): 616.29 [M+H]^+^.

##### Characterization of 5,5′-(1-hexadecyl-1*H*-1,2,3-triazole-4,5-diyl)bis(4-phenyl-2,4-dihydro-1,2,4-triazole-3-thione) (**6d**)

It was obtained in 85% as colorless crystals, mp: 250–251 °C. IR (KBr): 3368 (NH), 1610 (C=N), 1578 (C=C), 1299 cm^−1^ (C=S). ^1^H NMR (400 MHz, DMSO-*d*_*6*_): δ_H_ = 0.86 (t, 3H, *J* = 4 Hz, C**H**_3_), 1.23–1.28 (m, 22H, 11 × C**H**_2_), 1.34–1.44 (m, 4H, 2 × CH_2_), 1.84–1.88 (m, 2H, NCH_2_C**H**_2_), 4.16 (bs, 2H, NC**H**_2_), 7.02–7.49 (m, 10H, Ar–**H**), 10.60 (bs, 2H, 2 × **N**H). ^13^C NMR (100 MHz, DMSO-*d*_*6*_): δ_C_ = 14.63 (**C**H_3_), 22.77, 26.47, 28.00, 29.18, 29.37, 29.59, 29.69 (**C**H_2_), 31.96 (N**C**H_2_), 118.03, 123.22, 129.85, 130.64, 140.49, 187.84 (Ar–**C**, **C**=N, **C**=S). Anal. Calcd. For C_34_H_45_N_9_S_2_: C, 63.42; H, 7.04; N, 19.58. Found: C, 63.31; H, 7.11; N, 19.66. ESI MS (*m/z*): 644.32 [M+H]^+^.

#### Synthesis and characterization of 1,2,3-triazole bis-4-amino-1,2,4-triazole-3-thiones **7a**–**d**

Step 1: Carbon disulfide (30 mmol) was added dropwise to a stirred solution of compound **4a**–**d** (10 mmol) dissolved in absolute ethanol (50 mL) containing potassium hydroxide (30 mmol) at 0 °C. The stirring was continued for 16 h at ambient temperature, and then diluted with diethyl ether. The obtained precipitate was collected by filtration, washed with diethyl ether, dried to afford the corresponding potassium dithiocarbazinate salt and used without further purification as it was moisture sensitive.
Step 2: Hydrazine hydrate (30 mmol) was added to a solution of the potassium salt (10 mmol) dissolved in water (10 mL). The reaction mixture was then heated under reflux for 6 h. After cooling, the reaction mixture was acidified with HCl. The solid thus formed was collected by filtration, washed with water and recrystallized from ethanol to yield the desired aminotriazole **7a**–**d**.

##### Characterization of 5,5′-(1-decyl-1*H*-1,2,3-triazole-4,5-diyl)bis(4-amino-2,4-dihydro-1,2,4-triazole-thione) (**7a**)

It was obtained in 80% as colorless crystals, mp: 217–218 °C. IR (KBr): 3295–3350 (NH), 1611 (C=N), 1584 (C=C), 1288 (C=S) cm^−1^. ^1^H-NMR (400 MHz, CDCl_3_): δ_H_ = 0.90–0.93 (m, 3H, C**H**_3_), 1.25–1.41 (m, 14H, 7 × C**H**_2_), 1.78–1.84 (m, 2H, NCH_2_C**H**_2_), 4.20–4.27 (m, 2H, NC**H**_2_), 5.22 (bs, 4H, 2 × **N**H_2_), 7.13–7.41 (m, 10H, Ar–**H**), 9.21 (bs, 2H, 2 × **N**H). ^13^C NMR (100 MHz, CDCl_3_): δ_C_ = 14.15 (**C**H_3_), 15.27, 22.74, 26.34, 26.45, 28.80, 29.29, 29.33, 29.48, 30.01 (**C**H_2_), 31.88 (N**C**H_2_), 129.73, 137.38, 142.03, 187.63 (Ar–**C**, **C**=N, **C**=S). Anal. Calcd. For C_16_H_27_N_11_S_2_: C, 43.92; H, 6.22; N, 35.21. Found: C, 43.86; H, 6.10; N, 35.08. ESI MS (*m/z*): 438.18 [M+H]^+^.

##### Characterization of 5,5′-(1-dodecyl-1*H*-1,2,3-triazole-4,5-diyl)bis(4-amino-2,4-dihydro-1,2,4-triazole-thione) (**7b**)

It was obtained in 84% as colorless crystals, mp: 234–235 °C. IR (KBr): 3278–3340 (NH), 1608 (C=N), 1578 (C=C), 1291 (C=S) cm^−1^. ^1^H-NMR (400 MHz, CDCl_3_): δ_H_ = 0.86–0.90 (m, 3H, C**H**_3_), 1.25–1.39 (m, 18H, 9 × C**H**_2_), 1.83–1.89 (m, 2H, NCH_2_C**H**_2_), 4.21–4.30 (m, 2H, NC**H**_2_), 5.25 (bs, 4H, 2 × **N**H_2_), 7.09–7.41 (m, 10H, Ar–**H**), 9.25 (bs, 2H, 2 × **N**H). ^13^C NMR (100 MHz, CDCl_3_): δ_C_ = 14.11 (**C**H_3_), 15.21, 22.72, 22.98, 26.38, 26.62, 28.84, 29.01, 29.34, 29.53, 29.70, 29.94, 30.31 (**C**H_2_), 31.91 (N**C**H_2_), 129.78, 137.52, 141.43, 187.65 (Ar–**C**, C=N, C=S). Anal. Calcd. For C_30_H_37_N_9_S_2_: C, 61.30; H, 6.34; N, 21.40. Found: C, 61.36; H, 6.25; N, 21.34. ESI MS (*m/z*): 588.26 [M+H]^+^.

##### Characterization of 5,5′-(1-tetradecyl-1*H*-1,2,3-triazole-4,5-diyl)bis(4-amino-2,4-dihydro-1,2,4-triazole-thione) (**7c**)

It was obtained in 83% as colorless crystals, mp: 251–252 °C. IR (KBr): 3285–3340 (NH), 1620 (C=N), 1578 (C=C), 1290 (C=S) cm^−1^. ^1^H-NMR (400 MHz, CDCl_3_): δ_H_ = 0.91 (t, 3H, *J* = 8 Hz, C**H**_3_), 1.28-1.39 (m, 22H, 11 × C**H**_2_), 1.84–1.89 (m, 2H, NCH_2_C**H**_2_), 4.24–4.31 (m, 2H, NC**H**_2_), 5.19 (bs, 4H, 2 × **N**H_2_), 7.11–7.41 (m, 10H, Ar–**H**), 9.28 (bs, 2H, 2 × **N**H). ^13^C NMR (100 MHz, CDCl_3_): δ_C_ = 14.12 (**C**H_3_), 15.23, 22.74, 22.90, 26.39, 26.59, 28.82, 29.04, 29.38, 29.75, 29.84, 29.91, 30.21, 30.32 (**C**H_2_), 31.85 (N**C**H_2_), 129.75, 137.49, 141.46, 187.60 (Ar–**C**, **C**=N, **C**=S). Anal. Calcd. For C_32_H_41_N_9_S_2_: C, 62.41; H, 6.71; N, 20.47. Found: C, 62.36; H, 6.65; N, 20.39. ESI MS (*m/z*): 616.29 [M+H]^+^.

##### Characterization of 5,5′-(1-hexadecyl-1*H*-1,2,3-triazole-4,5-diyl)bis(4-amino-2,4-dihydro-1,2,4-triazole-thione) (**7d**)

It was obtained in 85% as colorless crystals, mp: 275–276 °C. IR (KBr): 3275–3350 (NH), 1615 (C=N), 1580 (C=C), 1296 cm^−1^ (C=S). ^1^H NMR (400 MHz, CDCl_3_): δ_H_ = 0.85–0.91 (m, 3H, C**H**_3_), 1.25–1.29 (m, 22H, 11 × C**H**_2_), 1.36–1.43 (m, 4H, 2 × CH_2_), 1.85–1.90 (m, 2H, NCH_2_C**H**_2_), 4.19 (bs, 2H, NC**H**_2_), 5.27 (bs, 4H, 2 × **N**H_2_), 7.08–7.43 (m, 10H, Ar–**H**), 9.31 (bs, 2H, 2 × **N**H). ^13^C NMR (100 MHz, DMSO-*d*_*6*_): δ_C_ = 14.63 (**C**H_3_), 15.30, 22.70, 22.92, 26.35, 26.62, 28.77, 29.01, 29.42, 29.70, 29.82, 29.95, 30.17, 30.28 (**C**H_2_), 31.90 (N**C**H_2_), 129.70, 137.44, 141.51, 187.68 (Ar–**C**, **C**=N, **C**=S). Anal. Calcd. For C_34_H_45_N_9_S_2_: C, 63.42; H, 7.04; N, 19.58. Found: C, 63.31; H, 7.11; N, 19.69. ESI MS (*m/z*): 616.29 [M+H]^+^.

### Antimicrobial activity assay

Determination of minimum inhibitory concentration (MIC) was conducted according to the microdilution method [36], as previously described. The newly designed compounds were evaluated for their antimicrobial activity against six pathogenic bacterial strains [Gram-positive: *Bacillus cereus* (ATTC 10876), *Enterococcus faecalis* (ATTC 29212) and *Staphylococcus aureus* (ATTC 25923), Gram-negative: *Proteus mirabilis* (ATTC 35659), *Escherichia coli* (ATTC 25922) and *Pseudomonas aeruginosa* (ATTC 27853), and two fungal strains (*Candida albicans* (ATTC 50193) and *Aspergillus brasiliensis* (ATTC 16404)].

MIC tests were undertaken in 96 flat bottom microtiter plates (TPP, Switzerland). An inoculum size of 1 × 10^5^ CFU mL^−1^ of each microorganism was inoculated in each microtiter plate well. Test wells were filled with 100 μL nutrient broth and a series of dilutions of each examined compound dissolved in DMSO (1–500 mg mL^−1^). Positive control wells consisted of the individual microorganism under investigation inoculated in 100 μL nutrient broth while negative control wells contained DMSO at the same concentration present in the test wells.

Plates were incubated for 24 h at 37 °C, with shaking. To evaluate microbial growth, optical densities were measured at 600 nm (OD600) using a Microplate Reader (Palo Alto, CA, USA). The MIC value was designated as the least concentration at which more than 80% of the microbial growth is inhibited. MIC assessment was carried out in triplicates and repeated three times for each microorganism.

### In-silico molecular docking studies

The compounds synthesized in the present investigation were subjected for molecular docking studies using Auto Dock (version 4.0) with Lamarckian genetic algorithm [38]. We have considered using Lamarckian genetic algorithm over Monte Carlo simulated annealing and traditional genetic algorithm. The previous method can handle ligands with more degrees of freedom than the Monte Carlo method used in earlier versions of AUTODOCK. The Lamarckian genetic algorithm is the most efficient, reliable, and successful. AutoDock 4.0, combines energy evaluation through grids of affinity potential employing various search algorithms to find the suitable binding position for a ligand on a given protein. The ligands were drawn in ChemSketch. Energy of molecule was minimized using by PRODRG server [39]. In the present study, the binding site was selected based on the amino acid residues, which are involved in binding with glucosamine-6-phosphate of GlcN-6-P synthase as obtained from Protein Data Bank (http://www.pdb.org/pdb/home/home.do) with the PDB ID 2VF5 which would be considered as the best accurate active region as it is solved by experimental crystallographic data [40]. It was then edited by removing the heteroatoms, adding the C-terminal oxygen, rotating all the torsions during docking. Steepest Descent methods were applied for minimization by considering the default parameters. Polar hydrogen’s were added to ligands using the hydrogen’s module in Autodock tool and thereafter assigning Kollman united atom partial charges. Docking to ligands was carried out with standard docking protocol on the basis a population size of 150 randomly placed individuals; a maximum number of 2.5*107 energy evaluations, a mutation rate of 0.02, a crossover rate of 0.80 and an elitism value of 1. Fifteen independent docking runs were carried out for ligands. The grid was centered at the region including all the 12 amino acid residues (Ala602, Val399, Ala400, Gly301, Thr302, Ser303, Cys300, Gln348, Ser349, Thr352, Ser347 and Lys603). The grid box size was set at 70, 64, and 56 Å̊ for x, y and z respectively, and the grid center was set to 30.59, 15.822 and 3.497 for x, y and z respectively, which covered all the 12 amino acid residues in the considered active pocket. The spacing between grid points was 0.375 angstroms. The docking results were interpreted according to the.pdb file. Using the rmsd table created in the.dlg file, we have determined the co-ordinates of the minimum energy run. UCSF chimera was used to visualize the coordinate of the docked protein along with targeted compounds within 6.5 Ǻ region.

## Conclusions

A series of novel 1,2,3-triazole-1,2,4-triazole hybrids carrying variant lipophilic side chain were synthesized and screened for antibacterial and antifungal activity. Finally, the synthesized compounds were docked inside the active site of Glucosamine-6-phosphate synthase, the potential target for antimicrobial and antifungal agents and the results of such studies were reported. In-silico studies revealed that all the synthesized compounds **3a**, **c**, **d**, **4a**–**d**, **5a**–**c**, **6a**–**d**, **7a**–**d** have relatively less binding energy as compared to the standard drug and may be considered as a good inhibitors of GlcN-6-P. The binding energy toward the target protein ranged from − 5.72 to − 10.49 kJ mol^−1^. The high-ranking binding energy of the synthesized compound, **6d** was − 10.49 kcal/mL. Consistent with the in silico studies, all synthesized compounds demonstrated fair to excellent antimicrobial activities relative to standard potent antibacterial and antifungal agents, with remarkably enhanced antimicrobial activities associated with the 1,2,4-triazole derivatives tailoring elongated chain substitution at the 1,2,3-triazole N-1 position.
